# Retinal proteomics in neurodegeneration: Insights into ocular and brain disorders

**DOI:** 10.4103/NRR.NRR-D-25-00291

**Published:** 2025-10-30

**Authors:** Shahab Mirshahvaladi, Bhakta Prasad Gaire, Sara Assar Kashani, Aparajeeta Guha, Yosef Koronyo, Dieu-Trang Fuchs, Yuyi You, Keith L. Black, Joao A. Paulo, Stuart L. Graham, Vivek Gupta, Mehdi Mirzaei, Maya Koronyo-Hamaoui

**Affiliations:** 1 Macquarie Medical School, Faculty of Medicine, Health and Human Sciences, Macquarie University, Sydney, NSW, Australia; 2 Department of Neurosurgery, Maxine Dunitz Neurosurgical Research Institute, Cedars-Sinai Medical Center, Los Angeles, CA, USA; 3 MND Research Centre, Macquarie Medical School, Faculty of Medicine, Health and Human Sciences, Macquarie University, Sydney, NSW, Australia; 4 Save Sight Institute, The University of Sydney, Sydney, NSW, Australia; 5 ProHeme Diagnostics Pty Ltd, Sydney, NSW, Australia; 6 Department of Cell Biology, Harvard Medical School, Boston, MA, USA; 7 Department of Biomedical Sciences, Division of Applied Cell Biology and Physiology, Cedars-Sinai Medical Center, Los Angeles, CA, USA; 8 Department of Neurology, Cedars-Sinai Medical Center, Los Angeles, CA, USA

**Keywords:** age-related macular degeneration, biomarker discovery, diabetic retinopathy, glaucoma, mechanism of disease, neurodegeneration, post-translational modification, proteomics, retinal proteome, retinitis pigmentosa

## Abstract

Dysregulated proteome in the retina represents a promising avenue for discovering novel therapeutic targets and noninvasive diagnostic biomarkers for neurodegenerative diseases with ocular manifestations. Advanced mass spectrometry–based proteomics techniques have shown considerable potential in investigating the retinal proteome in diseases such as glaucoma, age-related macular degeneration, diabetic retinopathy, retinitis pigmentosa, as well as Alzheimer’s disease, amyotrophic lateral sclerosis, and Parkinson’s disease. Recent proteomics innovations are overcoming challenges such as limited sample size and protein coverage that previously hindered comprehensive retinal proteome analysis. Notably, the incorporation of artificial intelligence–driven computational pipelines, including Graphics Processing Unit-accelerated deep learning architectures, has markedly enhanced the precision and effectiveness of retinal proteomics. These advances facilitate high-resolution identification of novel protein signatures within large-scale multi-omics datasets. Furthermore, the integration of advanced artificial intelligence with state-of-the-art big data infrastructures supports the early detection of biomarkers and therapeutic targets in neurodegenerative diseases with ocular involvement, offering unprecedented disease specificity and sensitivity. In addition to these computational strides, emerging complementary and alternative technologies continue to provide valuable tools for retinal analysis, expanding the potential for identifying biomarker and therapeutic targets in both ophthalmic and neurodegenerative disorders. This review summarizes recent advancements in retinal proteomics, with a particular focus on neurodegenerative and ocular diseases.

## Introduction

Over the past two decades, genome-wide association studies have linked numerous genomic alterations to various diseases (Chong et al., 2015). However, in complex diseases such as glaucoma, more than hundreds of genes can be involved, making it difficult to pinpoint therapeutic targets (Andrews et al., 2023). This challenge has underscored the need for a more holistic systems biology approach, one that integrates genomics, transcriptomics, and especially proteomics to identify novel targets and biomarkers (Geschwind and Konopka, 2009; Rollo et al., 2016) in the “big data” era.

Unlike genes or transcripts, proteins are not static as their abundance levels or modifications change as a function of cellular development, aging, and disease. Accurate protein measurement techniques in different cells and tissues are thus crucial for understanding disease biology. The term ‘proteome’ is the collection of all proteins found within the cell or tissue, mirroring the functional status of that biological system. Accordingly, proteomics is the large scale study of the proteome, provides deeper insight into molecular function under physiological and disease (Sinha and Mann, 2020). As a key requirement of systems biology, understanding the proteome is vital, as protein machinery carries out the bulk of cellular processes, including signaling, metabolism, and structural integrity (Bludau and Aebersold, 2020). A glossary of key proteomics methodologies referenced in this review is provided in **Table 1**.

**Table 1:** Glossary of key proteomics terms used in this review

Term	Definition
LC–MS	A common analytical technique in proteomics that couples liquid chromatography (LC) with mass spectrometry (MS) to separate and analyze proteins. LC separates peptides based on their chemical properties while MS detects and measures the mass-to-charge (m/z) ratios (Fenn et al., 1989).
LC–MS/MS	A technique that combines LC for separating complex peptides with tandem mass spectrometry (MS/MS) for identifying proteins based on m/z ratios and fragmentation patterns (Eng et al., 1994).
Two-dimensional gel electrophoresis	Separates proteins by isoelectric point in the first dimension and molecular weight in the second; commonly followed by staining and spot analysis (O'Farrell, 1975).
Shotgun proteomics	Proteins are enzymatically digested into peptides before mass spectrometry analysis, enabling identification of thousands of proteins in complex samples without prior separation (Washburn et al., 2001).
Label-free quantification	Quantifies relative protein abundances without the use of stable isotope labelling, based on peptide signal intensities or spectral counting (Cox et al., 2014).
Isobaric tags for relative and absolute quantification (iTRAQ)	Chemical labelling method using isobaric tags to label peptides from different samples, enabling simultaneous protein identification and quantification (Ross et al., 2004).
Tandem mass tag	A chemical labelling approach similar to iTRAQ, enabling multiplexed quantitative analysis of proteins from multiple samples in a single MS run (Thompson et al., 2003).
Data-dependent acquisition	A method where the most abundant precursor ions are selected for fragmentation and MS/MS analysis in real time, providing targeted information for selected peptides (Olsen et al., 2007).
Data-independent acquisition	A method that fragments all precursor ions across selected m/z ranges in each cycle, capturing comprehensive peptide fragmentation data for unbiased analysis (Gillet et al., 2012).

Exploring the dynamic proteome is a fundamental quest in biology as this network is constantly responding to internal and external stimuli, shaping the tissue identity and its observable phenotype. Moreover, proteins comprise the majority of pharmaceutically active components and almost all drugs target, making proteomics central to drug discovery and therapeutic applications (Aggarwal and Lee, 2003; Butcher et al., 2004). One of the goals of proteomics is to identify biomarkers and therapeutic targets, enabling early detection and personalized medicine (Shi et al., 2009).

By definition, neurodegenerative diseases are a diverse category of conditions marked by the gradual and progressive loss of neurons. This process often involves the preferential vulnerability of specific nerve cell populations, resulting in distinct neurological symptoms (Wareham et al., 2022). So far, mass spectrometry-based proteomics has proved a powerful tool for addressing knowledge gaps in neurodegeneration (Bai et al., 2021) and in particular, retinal neuropathies (Cehofski et al., 2017). Proteomics is particularly well-suited for studying neurodegenerative conditions as these diseases often involve proteinopathies, including abnormal expression, aggregation, degradation, or aberrant post-translational modifications (PTMs), all of which cannot be fully captured by genomics or transcriptomics alone (Schaffert and Carter, 2020; Wilson et al., 2023).

Among neurodegenerative diseases, retinal neuropathies provide a unique and accessible model for noninvasive, high-resolution live imaging. Retinal neurodegeneration is a leading cause of irreversible vision loss in developed countries (Punzo et al., 2012; Flaxman et al., 2017; Mutlu et al., 2018; Garzone et al., 2023; Sun et al., 2023), and is increasingly recognized as a feature of neurodegenerative diseases such as Alzheimer’s disease (AD) (Chang et al., 2014; Cunha et al., 2016; Salobrar-Garcia et al., 2019; Javitt et al., 2023). While affecting distinct parts of the retina, these diseases share common underlying mechanisms, often insidious and asymptomatic at early stages and ultimately lead to significant visual impairment. The complex and multifactorial nature of these diseases, driven by both genetic and environmental factors, presents a complex burden for the health system (Muc et al., 2018). By analyzing the complex proteomes of retina in various states, researchers have provided invaluable insights into the potential molecular mechanisms driving ocular and neurodegenerative diseases (Tezel, 2014).

**Figure 1:**
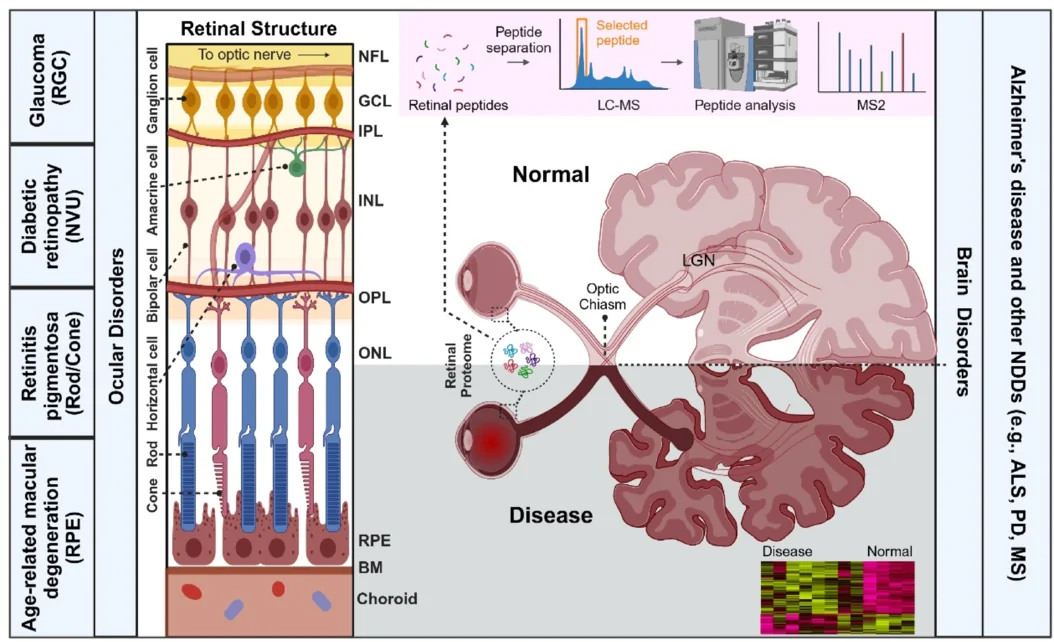
Schematic composition of retinal cells/layers and their connection to neurodegenerative diseases discussed in this review. Heatmap in the lower right corner depicts an example of up-regulated (pink) and downregulated (green) proteins in normal and diseased retina analyzed through mass spectrometry. Created with BioRender.com and licensed under BioRender’s Academic License g92x881. ALS: Amyotrophic lateral sclerosis; BM: Bruch’s membrane; GCL: ganglion cell layer; INL: inner nuclear layer; IPL: inner plexiform layer; LC-MS: liquid chromatography–mass spectrometry; LGN: lateral geniculate nucleus; MS: multiple sclerosis; MS2: tandem mass spectrometry; NDDs: neurodegenerative diseases; NFL: nerve fiber layer; NVU: neurovascular unit; ONL: outer nuclear layer; OPL: outer plexiform layer; PD: Parkinson’s disease; RGC: retinal ganglion cell; RPE: retinal pigment epithelium.

As new proteomics technologies continue to advance, we can anticipate further breakthroughs in our understanding of neurodegenerative eye diseases (Tezel, 2014). One of the examples of such systematic initiatives is the EyeOME project (Ahmad et al., 2018), which seeks to provide proteomics insights into ophthalmologic and retinal disorders as part of Human Proteome Project (Omenn et al., 2024). This review synthesizes proteomic landscape of the retina in major neurodegenerative ocular and brain disorders (**Figure 1**), with a focus on comparing protein signatures and implicated pathways across these disorders. Building on this foundation, we highlight how integrating proteomics with new tools enables the translation of these discoveries into clinical practice.

## Search Strategy

A literature search was conducted using PubMed and Google Scholar databases. Searches were performed between September and December 2024, covering English full-text articles from 2014 to 2024. The terms included combinations of the following keywords: “retina,” “mass spectrometry,” “proteomics,” “glaucoma,” “age-related macular degeneration,” “diabetic retinopathy,” “retinitis pigmentosa,” “Alzheimer’s disease,” “neurodegeneration,” and “posttranslational modification.” Boolean operators (AND, OR) were used to combine search terms. Inclusion criteria encompassed original research articles using various proteomics methodologies that focused on retinal proteomics in the context of ocular or neurodegenerative diseases. The “Review” filter was also used to capture key prior reviews. We excluded articles focusing solely on *in vitro* studies, conference abstracts without full-text availability, and studies that did not involve proteomics analysis. Additional relevant articles were identified using the citation-tracking tool ResearchRabbit (Cole and Boutet, 2023) and by reviewing the reference lists of selected articles. For each disease category, we prioritized studies that provided comprehensive proteomic profiling and identified novel biomarkers or therapeutic targets.

## Retinal Structure and Central Nervous System Connection

The human eye is part of the sensory nervous system and serves as the organ responsible for light perception and vision. Lacking the current knowledge of the structure of the retina, Charles Darwin expressed skepticism that natural selection could have produced such a complex organ as the eye, deeming it “absurd in the highest possible degree” (Darwin, 1859). Although the mammalian retina contains over 60 types of neurons, it is composed of six main neuronal cell types and one type of glial cell, organized into three layers that are highly conserved across vertebrates: photoreceptors (rods and cones), retinal pigment epithelium (RPE), bipolar cells, amacrine cells, horizontal cells, Müller cells and retinal ganglion cells (RGCs) (Seung and Sümbül, 2014). Light captured by photoreceptors is transmitted by bipolar cells as signals that trigger a cascade of events, altering membrane potential and neurotransmitter release at synapses in the outer plexiform layer. These events initiate a series of signals that reach the RGCs.

The axons of RGCs form the optic nerve (ON) that extends to the lateral geniculate nucleus in the thalamus and the superior colliculus in the midbrain, where these information is relayed to higher visual processing centers to perceive the image (Field and Chichilnisky, 2007; Hoon et al., 2014; **Figure 1**). Additionally, some optic nerve fibers project to midbrain structures such as the superior colliculus, which in important in visual reflexes, and the suprachiasmatic nucleus of the hypothalamus that is responsible for regulating circadian rhythms (Prasad and Galetta, 2011). Due to high metabolic demand and susceptibility to damage from oxidative stress, inflammation, ischemia, and mitochondrial dysfunction, the ON is particularly vulnerable in neurodegenerative conditions (Carelli et al., 2017). Consequently, its degeneration can contribute to vision loss in conditions such as glaucoma, multiple sclerosis-associated optic neuritis, ischemic optic neuropathy, and traumatic optic neuropathy (You et al., 2013; Levin, 2018). Given its anatomical accessibility, optic nerve pathology provides valuable insights into broader diseases, making it an important model for studying neurodegeneration and testing therapeutic strategies (Mancino et al., 2019; Wareham et al., 2022; Koronyo et al., 2023). Despite significant advances in proteomic technologies and their widespread application in studying neurodegenerative diseases, the ON has remained relatively understudied in the context of proteomics. For detailed reviews on proteomics of ON in neuropathies, refer to (Tezel, 2016; Meehan et al., 2021; Meng et al., 2023; Prokai et al., 2023).

The notion that the eyes are the window to the soul was notably explored by Marcus Cicero and later Plato. However, a more scientific description is the analogy of retina to the central nervous system (CNS). Mammalian retina shares similar anatomy, functional elements, and response to insults with CNS. Thus, as the peripheral part of the nervous system not shielded by bone and directly observable without invasive procedures, the retina offers a unique opportunity to study structure and function of the brain as its integral part (Koronyo-Hamaoui et al., 2011; London et al., 2013; Hart et al., 2016; Gaire et al., 2024). Similar to other CNS axonal injuries, insults such as excitotoxic neurotransmitter levels, oxidative stress, aggregated proteins or neurotrophic factor deprivation can trigger scar formation, myelin destruction, and retrograde/anterograde degeneration of axons (Schwartz et al., 1996; Wang and Mao, 2021; Boccuni and Fairless, 2022).

## Glaucoma

Glaucoma, a leading cause of irreversible vision loss, encompasses various conditions with diverse presentations and is characterized by the progressive degeneration of RGCs, resulting in optic nerve damage. Prior to this cell death, axonal atrophy and impaired axonal transport are observed (Buckingham et al., 2008; Calkins, 2008). The precise causes of glaucoma and similar degenerative ocular diseases still remain unclear, while both genetic and environmental factors influence their onset (Schmidt et al., 2008). Elevated intraocular pressure (IOP) is associated with glaucoma risk (Leske et al., 2001), though it is not its sole cause (Le et al., 2003). While many people with high IOP do not develop the disease, increased IOP remains a significant risk factor (Shazly and Latina, 2009). Currently, IOP reduction is the only modifiable risk factor for glaucoma, and more than 9% of patients with untreated ocular hypertension develop glaucoma within five years. Lowering IOP has been shown to slow disease progression and cut this rate in half (Kass et al., 2002)

In primary open-angle glaucoma, resistance to aqueous humor outflow in the trabecular meshwork elevates IOP (Weinreb et al., 2016). In angle-closure glaucoma, iris obstruction limits access to trabecular meshwork drainage (Congdon and Friedman, 2003). Primary angle-closure glaucoma is another form of this condition, which is more common in Asian populations and considered more aggressive (Sun et al., 2017). Primary angle-closure glaucoma can result from abnormalities in the iris, lens, or retrolenticular structures, and non-pupil block mechanisms are known to cause angle closure in some Asian patients (Gao et al., 1989). Intriguingly, around one-third of glaucoma cases are classified as normal-tension glaucoma (NTG), where elevated IOP is not reported (Gutteridge, 2000). Optic nerve damage in NTG may be caused by changes in the translaminar pressure difference driven by shifts in cerebrospinal fluid-mediated intracranial pressure (Jonas et al., 2015). Low intracranial pressure and elevated IOP are thought to impact the lamina cribrosa through similar mechanisms, with studies indicating lower intracranial pressure in NTG patients (thus elevated translaminar pressure difference) compared to those with high-tension glaucoma and healthy controls (Berdahl et al., 2008; Ren et al., 2010).

Before the introduction of gel-free methods, two-dimensional gel electrophoresis (2-DE) or difference gel electrophoresis were the common approaches to investigate protein changes in glaucoma (Marcus et al., 2020). Early studies identified proteins involved in glycolysis, stress response, and excitotoxicity, in retinal lysates from ocular hypertensive rat eyes compared with the controls (Tezel et al., 2005). Later studies using the DBA/2J mouse model revealed changes in proteins such as integrin β7, suggesting a role in RGC death (Kanamoto et al., 2009). Schallenberg et al. (2012) used a rat model of ocular hypertension and used travaprost and dorzolamide in the form of eyedrop to lower IOP. Proteins such as high mobility group box 1, heat shock protein 70, calmodulin, and carbonic anhydrase II were identified by liquid chromatography-tandem mass spectrometry (LC–MS/MS) and suggested as potential biomarkers or therapeutic targets (Schallenberg et al., 2012).

Recent proteomic approaches have extensively progressed our understanding of molecular mechanisms in glaucoma, with Funke et al. (2017) providing a comprehensive overview of protein alterations and emerging biomarkers in this disease. For over two decades, gel-free proteomics methods have revolutionized retina proteomics in glaucoma research. Label-free shotgun proteomics, for example, enabled the identification of hundreds of proteins in early experimental glaucoma model in rhesus macaques and the analysis of protein expression changes in response to chronic IOP elevation and optic nerve transection. This approach revealed the dynamic nature of the retinal proteome and identified potential mechanisms of early retinal response to chronic IOP elevation, such as cytoarchitecture regulation (Stowell et al., 2011). Additionally, quantitative proteomics using Isobaric Tags for Relative and Absolute Quantitation (iTRAQ) uncovered the neuroprotective effects of growth factors such as hepatocyte growth factor on injured RGCs following optic transection. Their findings indicated that hepatocyte growth factor rescued RGCs through both mitogen-activated protein (MAP) kinase and phosphoinositide 3-kinase in the rat retina (Hollander et al., 2012).

Using shotgun 2-DE and oxygen isotope labeling for relative quantification of protein expression, Yang et al. identified more than 2000 proteins, of which hundreds showed dysregulation in the retinas of individuals with ocular hypertension, compared to normotensive controls. Bioinformatics analysis revealed hypertension–induced early changes involved various pathways such as cellular homeostasis, heat shock proteins, ubiquitin proteasome system, antioxidant proteins, DNA repair enzymes, as well as oxidative phosphorylation proteins. Notably, these changes occurred in the absence of detectable neuroinflammation or cell death (Yang et al., 2015). A label-free quantitative (LFQ) shotgun proteomics study identified over 600 proteins in the human retina and introduced new candidates such as adenine nucleotide translocator 3, DFS70, and MeCp2, mostly associated with mitochondrial dysfunction, stress proteins, molecular transport, and apoptosis pathways (Funke et al., 2016). Another LFQ study investigated the neuroprotective effects of α-crystallin B in occlusion of episcleral veins model of glaucoma. Intravitreal injection of recombinant α-crystallin B at the time of IOP elevation significantly protected the retina, as evidenced by improved visual function, retinal nerve fiber layer thickness, and RGC counts in rats. Functional enrichment analysis revealed upregulation of all α, β, and γ subclasses of the crystallin protein family, suggesting a broader protective effect of α-crystallin B in glaucoma (Anders et al., 2017).

Another LFQ proteomics study investigates the role of microglia in retinal degeneration triggered by intravitreal S100B injection in rats. Minocycline, a microglial inhibitor, was used to assess the impact of microglia on RGCs and optic nerve degeneration. Results showed that microglial inhibition provided partial protection to RGCs and optic nerve fibers, reducing inflammation, oxidative stress, and mitochondrial dysfunction. Proteomics analysis revealed that S100B-induced metabolic disturbances and inflammation contributed to degeneration, suggesting that microglial activation exacerbates degeneration but is not the sole cause of apoptosis in RGCs (Grotegut et al., 2020).

Similarly, Reinehr et al. (2021) used DIA-based LC–MS/MS analysis to explore time-dependent protein expression changes in an experimental autoimmune glaucoma using S100B immunization in rats. The study identified more than 1700 proteins with high confidence, of which 43 and 67 proteins were significantly altered after one week and two weeks post-immunization, respectively. Proteins such as α2-macroglobulin and heat shock protein 60 were upregulated in the retina, highlighting the role of the immune system associated with disease progression in experimental autoimmune glaucoma model of NTG (Reinehr et al., 2021). Another recent DIA MS/MS study mapped the proteome of degenerative and regenerative mouse retinas after elevated intraocular pressure IOP, identifying over 5700 proteins. Regenerative condition was linked to thyroid hormone, Notch, and Wnt signaling, while degenerative condition shared pathways such as mRNA surveillance and PD-L1 signaling. Common proteins, such as EP300 and nuclear factor-κB, highlight potential biomarkers for neuro-regeneration in glaucoma (Wang et al., 2024).

Using label-free SWATH MS/MS, Kwong et al. (2023) mapped the temporal proteomic changes associated with primary and secondary RGC loss following partial optic nerve transection rat model of glaucoma. They identified more than 2500 proteins, with 10, 25, and 61 differentially expressed proteins (DEPs) in the temporal retina at 1-, 4-, and 8-week post-injury, respectively. Notable findings included decreased ALDH1A1 and SNCG levels correlating with axonal loss and elevated glial fibrillary acidic protein, indicating regional astrocyte and Müller cell reactivity (Kwong et al., 2023).

In another study, the neuroprotective effects of 17β-estradiol (E2) in a rat glaucoma model with elevated IOP were investigated using LC-MS/MS with isotope-dilution quantification and selected reaction monitoring. Daily E2 eye drops significantly reduced RGC death and visual function loss, even with persistent high IOP. Quantitative proteomic analysis revealed upregulation of neuroprotective proteins such as α-crystallins and β-crystallin b2 associated with retinal health (Prokai-Tatrai et al., 2013). A similar work used targeted proteomics to study the prodrug 10β,17β-dihydroxyestra-1,4-dien-3-one (DHED) in a male rat model of glaucoma. DHED selectively converts to E2 in the retina after topical application, making it a safe neuroprotective candidate in ocular hypertension model of glaucoma. Using parallel reaction monitoring, the study analyzed a predefined panel of protein biomarkers associated with ocular hypertension-induced neurodegeneration. This targeted proteomic approach revealed that DHED-derived E2 corrected glaucomatous dysregulations in these biomarkers, preserving visual and structural integrity without systemic E2 elevation. In summary, these findings suggest the safety and efficacy of DHED for clinical glaucoma management (Kapic et al., 2024).

By utilizing tandem mass tag (TMT) technology, our group analyzed retinal tissues and vitreous humor from glaucoma patients and age-matched controls. Approximately 5000 proteins were quantified, with over 120 proteins identified as differentially expressed and linked to AD pathology. Key proteomic findings included mitochondrial dysfunction, evidenced by alterations in oxidative phosphorylation proteins, and activation of the innate immune response, as indicated by enrichment of classical complement pathway proteins. Moreover, many dysregulated proteins showed functional interaction networks implicated in AD and other neurodegenerative diseases, suggesting shared molecular mechanisms between glaucoma and these conditions (Mirzaei et al., 2017).

In another experiment using TMT-quantitative proteomics, Liu et al. (2021b) identified approximately 6300 proteins to understand the role of aging in the NTG mouse model. The identified proteins were associated with dysregulation of protein synthesis, age-dependent energy defects, and autophagy-lysosome pathway dysfunction. Certain biological features, including amyloid deposition, RNA splicing, microglia activation, and reduced crystallin production, were similar to those observed in AD. Several proteomic signatures that overlapped with retinal changes in the NTG mouse model were also found in the AD mouse model, suggesting common mechanisms between age-related degenerative disorders. These findings highlight potential new targets for diagnostic and therapeutic strategies. In a similar work, Li et al. (2022) revealed that TPM1, a pro-aging factor associated with functional deficits in normal aging retinas, is a potential systemic molecule underlying structural and functional deficits in the aging retina. Administration of recombinant TPM1 protein accelerated glial cell activation, dendritic sprouting of rod-bipolar and horizontal cells, and functional decline in the retinas of young mice. Conversely, treatment with an anti-TPM1 neutralizing antibody ameliorated age-related structural and functional changes in the retinas of aged mice. These findings suggest that targeting TPM1 could be a therapeutic strategy for mitigating age-related retinal degeneration.

In a later study using the TMT approach, our group analyzed retinal samples of a chronically elevated IOP model of glaucoma in rats, comparing findings with human glaucoma proteomics data. Over 4300 proteins with 248 differentially expressed were quantified, and key pathways such as mitochondrial dysfunction, oxidative phosphorylation deficits, glutathione metabolism, cytoskeletal alterations, and oxidative stress response were linked to glaucoma. Shared mechanisms between rat and human glaucoma include downregulation of crystallins and proteins involved in glutathione synthesis, while complement activation and cholesterol transport pathways were unique to human retina. These findings underline the role of oxidative stress, mitochondrial impairment, and cytoskeletal disruption in glaucoma pathogenesis and demonstrate the application of proteomics in elucidating neurodegenerative mechanisms and identifying novel therapeutic targets (Mirzaei et al., 2020).

A detailed summary of dysregulated proteins identified in proteomics studies on glaucoma is presented in **Table 2**. This compilation includes data from the past ten years covering both human donor tissues and a range of experimental glaucoma models, such as microbead injection, episcleral vein cauterization, autoimmune induction, and optic nerve crush. Each study entry highlights the proteomic method used, key upregulated/downregulated proteins, fold change thresholds, statistical significance (*P*-values or *Q*-values), and associated biological pathways. Collectively, these studies reveal consistent alterations in proteins involved in oxidative phosphorylation, immune activation, cytoskeletal remodeling, glial reactivity, and mitochondrial dysfunction, which are central to glaucoma pathophysiology.

**Table 2:** Summary of DEPs identified in proteomics studies on glaucoma

Source	Method	Key DEPs	FC	*P/Q*	Pathways	Reference
Human donors (ocular hypertension)	2-DE Shotgun	↑ CRYAA, DNAJC9, HSPA8, PSMA1, UBA1 ↓ ATP5C1, COX2, CYC1, NDUFA2, RHOT2, SDHB, VDAC1, VDAC2	≥2 or ≤0.5	*P* < 0.05	Heat shock proteins, ubiquitin proteasome, DNA repair, oxidative phosphorylation, complement system	Yang et al., 2015
Human Donor (glaucoma)	LFQ	↑ RALDH1, IRBP, HMGB1, VAPB, HSPB5, ANXA2, RTN3, GSTM3	≥1.4 or ≤0.71	*P* < 0.05	Cell death, oxidative reduction, stress response, immune system process	Funke et al., 2016
		↓ SLC25A6, NDUFB9, DFS70, Cl-B22, PDHE1, MECP2, ANT3, PSIP1				
Rat (episcleral vein cauterization-induced ocular hypertension)	LFQ	↑ CRYBA4, SUB1, CRYBB1, NUDT5, CLU, TOP2A ↓ INA, CSNK1D, RPS15, FGA, ADNP, ABCA2, DYRK1A	≥1.5 or ≤0.67	*P* < 0.05	Glial activation, cell-cycle control, apoptosis, oxidative stress, neurodegeneration	Anders et al., 2017
Human donors (open angle glaucoma)	TMT	↑ CLU, GSTM1, SRSF9, HNRNPM, C1q, C1, NDUFV3 ↓ GSTM1, CRYBA1, MRPL13	≥1.3 or ≤ 0.76	P ≤ 0.05	Oxidative phosphorylation, complement proteins, RNA processing, cholesterol metabolism	Mirzaei et al., 2017
Rat (S100B Autoimmune)	LFQ	↑ MSN, RHOT2, OLA1, POLD1 ↓ PTMS, CAMK2D, GAPDH, RPS17	NA	*P* < 0.05	Necrosis, apoptosis, reorganization of cytoskeleton, inflammation of organs, neurodegeneration	Grotegut et al., 2020
Rat (microbead-induced ocular hypertension)	TMT	↑ Car1, Hsd17b8, Calml3, Stat5a ↓ ATP6v1g1, GSS, Cryaa, Tpm1	≥1.2 or ≤0.83	P ≤ 0.05	Oxidative phosphorylation, actin filament, coagulation, RNA processing, redox regulation, SNARE complex machinery	Mirzaei et al., 2020a
Rat (S100B Autoimmune)	DIA	↑ α2-macroglobulin, Matrix Proteoglycans, IMPG1, PLS3 ↓ HSP60, MAP2	≥1.3 or ≤0.77	*P* < 0.05	Metabolism of carbohydrates, citric acid cycle, glutamate processing	Reinehr et al., 2021
Mouse (retrobulbar optic nerve crush)	LFQ	↑ GFAP, ALDH1A1, TRY10, ALB, HBB-B1 ↓ SNCG, NF-L, CDC42	≥1.4 or ≤0.71	*P* < 0.05	Neuron regeneration, response to wounding, eye development, peptidase activity,	Kwong et al., 2023
Rat (hyperosmotic saline-induced ocular hypertension)	DIA	↑ APOE, GNAO1, SNCG ↓ CNGB1, CRYAA, CRYBB, ATP1A1, HK2, ATP5F1B	NA	*P* < 0.05	Apoptotic pathway, mitochondrial dysfunction, chaperone-mediated autophagy signaling	Kapic et al., 2024
Mouse (episcleral vein cauterization-induced ocular hypertension)	LFQ	↑ CELF4, PPIG, TTHY, WBP2 ↓ RAP1A, GBB2, MGST3, K2C5	≥2or ≤0.5	*P* ≤ 0.05	Thyroid hormone, Notch, Wnt, VEGF signaling smRNA surveillance signaling, PD-1 checkpoint	Wang et al., 2024

This table compiles key dysregulated proteins published in the past ten years using human donor tissues or experimental models of glaucoma. Each entry includes the study, source of tissue, mass spectrometry method, key upregulated (↑) and downregulated proteins (↓) proteins, fold change, *P*-values or adjusted *P*-values (*Q*-values) and implicated biological pathways. 2-DE: Two-dimensional gel electrophoresis; DEPs: differentially expressed proteins; DIA: data-independent acquisition; FC: fold change; LFQ: label-free quantification; NA: not applicable; TMT: tandem mass tag; VEGF: vascular endothelial growth factor.

## Age-Related Macular Degeneration

Age-related macular degeneration is a leading cause of vision loss in older adults over 65 years of age. A hallmark of age-related macular degeneration (AMD) is the degeneration of the macula, often seen with extracellular deposits under the RPE termed druse. This accumulation of drusen as extracellular deposits between RPE basal membrane and Bruch’s membrane is a defining feature. Additionally, amyloid-beta (Aβ) protein has been reported in drusen, showing the increased risk of AMD by inducing oxidative stress, inflammation, and abnormal angiogenesis, comparable to its role in AD ((Johnson et al., 2002; Dentchev et al., 2003; Yoshida et al., 2005; Isas et al., 2010). The presence of multiple proteins in drusen, shared with Aβ plaques, suggests a potential role of protein misfolding in both diseases (Mullins et al., 2000; Luibl et al., 2006). This similarity between AMD and neurodegenerative disorders such as AD highlights potential shared therapeutic targets and a call for in-depth analysis of pathological mechanisms

Early AMD is often marked by basal linear deposits between these layers, a specific indicator of disease onset that leads to progressive macular function deterioration (Sarks et al., 2007). Lipofuscin buildups in the RPE, appearing as diffuse autofluorescent patches, are one of the earliest indicators of this condition (Kennedy et al., 1995). In the late-stage dry form, widespread RPE cell death and photoreceptor loss are observed (Sancho-Pelluz et al., 2008). In wet AMD, hypoxia-induced vascular endothelial growth factor secretion triggers choroidal neovascularization, which can result in subretinal or sub-RPE hemorrhages (Kulkarni and Kuppermann, 2005).

The RPE plays a crucial role in retinal function and disease, and its proteomic landscape has been comprehensively characterized in a comprehensive review, which summarizes key advances in RPE proteomics research (Beranova-Giorgianni and Giorgianni, 2018). The following paragraphs will highlight key findings in retina proteomics related to human donor samples and animal models of AMD.

In one of the first proteomics studies, Nordgaard et al. utilized 2-DE proteomics coupled with matrix-assisted laser desorption/ionization time-of-flight mass spectrometry analysis to identify RPE proteins from human donor eyes across four dry AMD stages. Early AMD stages showed reduced levels of heat shock proteins and apoptotic regulators, highlighting stress-induced protein misfolding, mitochondrial dysfunction, and apoptosis as potential causal mechanisms. In contrast, late-stage AMD was associated with changes in proteins involved in retinoic acid metabolism and rhodopsin regeneration, likely reflecting secondary disease effects (Nordgaard et al., 2006). In another study on human donor eyes using 2-DE approach, Ethen et al. identified 26 proteins with altered expression levels in human donor eyes during AMD progression. These proteins were linked to microtubule regulation and stress-induced protein protection and while approximately 60% were region-specific to the macula or periphery, approximately 40% changed in both regions, showing that AMD affects both macula and periphery in the retina (Ethen et al., 2006).

Although 2-DE is limited by its narrow dynamic range, low protein coverage, and bias toward soluble proteins, making it less suitable for comprehensive retinal proteome profiling compared to advanced methods such as LC-MS/MS, these early studies have provided valuable insights into the mechanisms of AMD. In a recent application of 2-DE, Karunadharma et al. (2022) aimed to differentiate between aging and AMD-associated changes and studied the RPE proteome of dry AMD and aging donors, compared with age-matched controls. They identified 58 proteins with altered expressions in which the aging group showed decreased metabolism, stress proteins, and proteases, while AMD group indicated increased glycolysis and fatty acid metabolism, decreased oxidative phosphorylation, and elevated stress and protein turnover. Their findings suggest AMD pathology involves distinct bioenergetic reprogramming and protein homeostasis disruptions beyond typical aging (Karunadharma et al., 2022).

Using LC–MS/MS-based shotgun proteomics, Crabb et al. (2002) investigated the composition of drusen and identified over 120 proteins in samples from dry AMD and normal donors. Key proteins such as tissue metalloproteinase inhibitor 3 and vitronectin were modified by oxidative damage, including carboxyethyl pyrrole and carboxymethyl lysine adducts, which were more abundant in AMD samples. These findings suggest oxidative injury plays a crucial role in drusen formation and AMD pathogenesis (Crabb et al., 2002). In a similar study, Umeda et al. (2005) characterized the molecular composition of drusen with early and late-onset macular degeneration in cynomolgus monkeys. Using shotgun approach, they showed that simian drusen shares similarities with human, including apolipoprotein E, complement proteins, annexins, and crystallins. Notably, autoantibodies, particularly against annexin II and μ-crystallin, were prevalent in affected monkeys, suggesting an autoimmune contribution to the disease (Umeda et al., 2005). Examining the composition of active retinal lipofuscin in dry AMD was performed using both 2-DE and shotgun proteomics by Warburton et al. (2005). They identified 41 proteins, mostly hydrophobic proteins and photoreceptors specific proteins such as rhodopsin inside these lipoprotein granules (Warburton et al., 2005). Finaly, Alcazar et al. (2009) analyzed the proteomic profile of RPE blebs using LC-MS/MS, identifying over 300 proteins involved in cell junctions, cytoskeletal regulation, oxidative phosphorylation, focal adhesion, and immune response. Notably, extracellular matrix remodeling proteins such as basigin and matrix metalloproteinase-14 were more prevalent in dry AMD patients (Alcazar et al., 2009).

In a quantitative proteomics study of the Bruch’s membrane of both dry and wet forms of AMD using iTRAQ, over 900 proteins were quantified, of which 56 were upregulated and 43 were downregulated. Immune response proteins, including complement components and damage-associated molecular patterns, were enriched in AMD samples. Retinoid processing proteins showed higher expression levels in early/mid AMD, while glycation-associated galectin-3 was upregulated in advanced dry AMD (Yuan et al., 2010). Distinct protein expression patterns were identified in wet AMD, including alterations in immune response and host defense mechanisms, suggesting that different pathways might be involved in the progression of wet AMD compared to dry AMD. Quantitative proteomics in large cohorts has been crucial for clinical research but faces challenges with reliability, accuracy, and reproducibility. Recently, Ultra High Resolution-IonStar has demonstrated significant accuracy, precision, and reproducibility, particularly for low-abundance proteins. Compared to SWATH-MS, this approach offered a higher accuracy in single-sample analyses within large cohorts, addressing a critical yet underexplored aspect of large-scale proteomic studies (Wang et al., 2021). Using this technology, Shen et al. analyzed human donor RPE samples graded for early AMD and age-matched controls to explore in-depth proteome dysregulations. More than 6000 unique proteins were quantified in 77 samples and pathways such as mitochondrial function, adenosine triphosphate (ATP) metabolism, lipid homeostasis, and oxidative stress exhibited dysregulation in dry AMD (Shen et al., 2023).

Lately, in an integrative multi-omics study, the genes and regulatory networks conserved in two mouse models of AMD and human samples were identified. Retinal RNA sequencing and TMT quantitative proteomics analysis in laser-induced choroidal neovascularization mouse model of wet AMD and NaIO3-induced retinal degeneration (dry AMD) revealed upregulated genes and pathways related to innate immunity and inflammation, particularly at early stages. Comparative analysis with AMD cohorts suggested 48 conserved inflammation-related proteins, including B2M, C3, and SERPING1, as the most consistent DEPs. These findings underscore the role of retinal inflammation in AMD pathogenesis and support the translational relevance of mouse models for studying AMD pathogenesis (Liu et al., 2024b).

In recent years, mass spectrometry-based proteomics has provided valuable insights into the molecular mechanisms underlying AMD. A detailed summary of dysregulated proteins identified in proteomics studies is presented in **Table 3**. This compilation covers research from the past ten years using both human donor tissues and animal models representing dry, early, and neovascular AMD. The studies employ various mass spectrometry approaches, including 2D-difference gel electrophoresis, label-free quantification, ultra-high-resolution strategies, and TMT-based workflows. Notably, proteins such as α-crystallins, ATP synthase subunits, and components of the tricarboxylic acid cycle and glycolysis pathways were frequently downregulated, suggesting early mitochondrial compromise. In contrast, proteomic analyses of neovascular AMD models highlighted the upregulation of inflammation- and angiogenesis-related markers, including CD44, STAT3, and ANXA2. These findings collectively point to a dynamic proteomic landscape in AMD that evolves from metabolic stress and impaired proteostasis in early stages to inflammatory and neovascular responses in late-stage disease. Together, these studies underscore the utility of retinal proteomics in identifying candidate biomarkers and therapeutic targets across the AMD spectrum.

**Table 3:** Summary of DEPs identified in proteomics studies on AMD

Source	Method	Key DEPs	FC	*P/Q*	Pathways	Reference
Human donors (dry AMD)	2-DE Shotgun	↑ INSR, RHOA, ACTB, ENO1, PGM1, PGK1, PKM, ALDH2 ↓ HMGCS2, HSP60, NDUFV2, PPA2, COX6B1, ATP5F1A	≥ 1.5 or ≤ 0.67	*P* ≤ 0.05	Metabolism, protein turnover, stress response, cell death, glycolysis, fatty acid metabolism, oxidative phosphorylation	Karunadharma et al., 2022
Human donors (early AMD)	UHR-IonStar	↑ KAIN, CATS, BT3A3, TPP2, GRAA ↓ PAR16, ORML3, ORML2, LSM12, ZN185	≥ 1.2 or ≤ 0.83	*P* ≤ 0.05	Translation, ATP metabolic process, lipid homeostasis, oxidative stress	Shen et al., 2023
Mouse (CNV model of AMD)	TMT	↑ CD44, STAT3, ANAX2, MSN, EZR, VIM, CD9, GFAP, CALM ↓ CNN3, MANF, SERPINH1, LAMP2, RG1, PON1, C3, A2M	≥ 1.2 or ≤ 0.83	*P* ≤ 0.05	Inflammation response, angiogenesis, immune effector process, epithelial morphogenesis, cytokine production	Liu et al., 2024a

This table compiles key dysregulated proteins published in the past ten years using human donor tissues or animal models. Each entry includes the study, source of tissue, mass spectrometry method, major upregulated (↑) and downregulated proteins (↓) proteins, fold change, *P*-values or adjusted *P*-values (*Q*-values) and implicated biological pathways. 2-DE: Two-dimensional gel electrophoresis; AMD: age-related macular degeneration; ATP: adenosine triphosphate; CNV: choroidal neovascularization; DEPs: differentially expressed proteins; FC: fold change; TMT: tandem mass tag.

## Diabetic Retinopathy

Diabetic retinopathy has been the primary cause of preventable blindness globally, predicted to affect 160 million patients by 2045 (Wong et al., 2016; Teo et al., 2021). Diabetes mellitus impacts various parts of the eye, but the main cause of blindness occurs in the retina. Traditionally, diabetic retinopathy has been viewed as a progressive microcirculatory disease of the retina in diabetic patients. However, recent evidence indicates that retinal neurodegeneration, including marked thinning of the neural retina, may be an early pathogenic event and could contribute to subsequent microvascular issues (Sohn et al., 2016).

For diabetic retinopathy, key risk factors include prolonged diabetes duration, poor glycemic control, and hypertension (Morello, 2007). Chronic hyperglycemia drives both neural and vascular changes in the retina. RGC apoptosis and glial cell activation lead to local inflammation, while vascular endothelial changes cause leukocytosis, which can obstruct blood vessels early in disease progression (Yu et al., 2015). Insulin dysregulation and the formation of advanced glycation end products are particularly involved in initiating retinal changes (Poulaki et al., 2004). Diabetes mellitus significantly raises the risk of visual impairment, with more than 8% of patients developing proliferative diabetic retinopathy (DR) and 24% developing diabetic macular edema within four years of diabetes mellitus onset. The longer the duration of diabetes, the greater the risk of developing DR, underscoring the importance of effective diabetes mellitus management to prevent vision-related complications. It is estimated that 10% of diabetes mellitus patients experience severe visual loss. Current therapies often address the advanced stages of DR, when vision impairment has already occurred (Simo and Hernández, 2014). DR is typically classified into non-proliferative and proliferative forms (Ting et al., 2016), with several histological markers, including gliosis and programmed cell death of neurons (Stem and Gardner, 2013).

Much of our current molecular understanding of DR mechanisms derives from experimental animal models, while knowledge about the biochemical changes in the diabetic retina prior to clinically observable symptoms remains limited. The loss of the blood-retinal barrier has been proposed as one of the most contributing factors, followed by leakage of plasma and retinal ischemia (Moss et al., 1998). Disruption in neurovascular coupling has been reported in both animal models (Mishra and Newman, 2010) and human patients (Nguyen et al., 2009). Diabetes mellitus also results in Müller glia swelling (McDowell et al., 2018) and activation of microglia, leading to further neuroinflammation and damage (Karlstetter et al., 2015). Eventually, the hypoxic conditions lead to angiogenesis, vitreous hemorrhage, and detachment of the retina (Wong et al., 2016).

Several proteomic studies have studied diabetic retinopathy in animal models. Using a streptozotocin-induced diabetes model, multiple studies have consistently observed upregulation of crystallin proteins and their normalization (Fort et al., 2009) or partial reversal (VanGuilder et al., 2011) by systemic insulin administration. This suggests a potential neuroprotective role for crystallins against inflammatory insults. In another study, Gao et al. (2009) analyzed the retinal proteome of diabetic C57BL/6 mice compared to age-matched controls and identified 65 DEPs, mostly involved in metabolism, oxidative phosphorylation, and apoptotic pathways. They observed that treatment with Candesartan, the blocker of angiotensin AT1 receptor, reversed over 72% of these protein changes in experimental diabetic retina.

Using iTRAQ, more than 340 DEPs were quantified in diabetic murine retinas, of which expression of 60 proteins involved in oxidative stress, energy metabolism, and apoptosis recovered to normal levels after phlorizin treatment (Zhang et al., 2013). This clearly demonstrates the broader applicability of proteomics, not only for identifying diagnostic biomarkers but also for screening the effects of novel therapeutics. In this case, phlorizin, a natural compound with anti-inflammatory and antioxidant properties, was used to treat DR mice.

A label-free shotgun proteomics study investigated the impact of metformin on the retinal proteome of *db/db* experimental diabetes mice. Bioinformatic analysis revealed decreased levels of proteins involved in synapse formation and suggested that metformin partially ameliorates diabetic retinopathy. Interestingly, VGLUT1 that loads glutamate neurotransmitter into synaptic vesicles, was identified as one of DEPs in animal models, without being reversed by metformin administration (Ly et al., 2014). Another label-free shotgun proteomics study has investigated the effects of placental growth factor ablation in C57BL/6 mice. The findings suggested the downregulation of insulin resistance proteins (G protein subunit beta 1, GNAI2, and GNAO1) and upregulation of PRDX6 and MAP2, involved in neuroprotective and antioxidant functions in placental growth factor deficient retinas (Saddala et al., 2018).

In a comparative study, Starr et al. (2024) induced RGC death in C57BL/6 mice using NMDA-mediated glutamate excitotoxicity and optic nerve crush. RGCs were isolated using CD90.2 MicroBeads and protein extracts were analyzed using LFQ LC-MS/MS for global proteomic profiling, identifying statistically significant changes in cellular signaling pathways. Notably, distinct (synaptic signaling) and overlapping (apoptosis) proteomic alterations were detected in both degenerative models, highlighting unique responses to excitotoxicity and mechanical damage. Their findings provide a comparative molecular framework for glutamate excitotoxicity and optic nerve crush, which are known to be involved in both glaucoma and diabetic retinopathy (Starr et al., 2024).

Lastly, a study conducted on 15 clinical samples detected over 2000 proteins in diabetic and nondiabetic postmortem retinas using gel-based LC/MS analysis. Remarkably, in diabetic retinas exhibiting glial activation pathways such as dopamine degradation and Parkinson’s signaling showed enrichment. However, diabetic retinas lacking glial activation showed enrichment in Aβ processing and neuregulin signaling pathways. This study reveals that DR and neurodegenerative diseases of the brain have shared pathogenic mechanisms (Sundstrom et al., 2018). New developments in the field of proteomics have enabled the analysis of murine (Quin et al., 2007; Fort et al., 2009; VanGuilder et al., 2011) and human retina samples (Zhang et al., 2015). Due to various challenges in obtaining human retinal tissues, vitreous fluid has been used frequently as an alternative to indirectly investigate early pathological events in the retina (Nakanishi et al., 2002; Garcia-Ramirez et al., 2007; Simó-Servat et al., 2012). Enhancing our understanding of the pathogenesis mechanisms of DR could pave the way for more effective early-stage prevention and intervention strategies, ideally before substantial microvascular damage and vision loss (Antonetti et al., 2012; Stitt et al., 2016).

A summary of dysregulated proteins identified in proteomics studies is presented in **Table 4**. This compilation encompasses proteomics research from the last decade, utilizing human donor retinal tissues and animal models of DR. These studies have employed various mass spectrometry techniques, enabling detection of proteomic alterations associated with hyperglycemia-induced retinal dysfunction. Notably, mitochondrial proteins and metabolic enzymes such as NDUFA9, ATP5L, and ACO2 were frequently upregulated, reflecting heightened metabolic activity and oxidative stress in diabetic retinae. Conversely, consistent downregulation was observed in cytoskeletal and synaptic proteins, including MAP2, TUBA1B, and SDHB, suggesting early synaptic compromise and impaired mitochondrial function. Several studies also reported upregulation of proteins linked to inflammatory signaling (e.g., vimentin and apolipoprotein E) and stress responses (e.g., superoxide dismutase 2 and FK506 binding protein 1A), while proteins involved in synaptic transmission, protein folding, and dopamine metabolism were notably reduced (Ly et al., 2014; Saddala et al., 2018; Sundstrom et al., 2018; Starr et al., 2024).

**Table 4:** Summary of DEPs identified in proteomics studies on diabetic retinopathy

Source	Method	Key DEPs	FC	*P/Q*	Pathways	Reference
Mouse (db/db model)	LFQ	↑ UQCRB, VAPA, GM4294, ROM1, UBB, SCAMP5, CDH2 ↓ GM6793, ATP6AP1, FUS, TUBB4A, SDHB, VDAC1	≥1.3 or ≤0.77	*P* ≤ 0.05	Synaptic transmission, catabolism of dopamine, cellular assembly, amino acid metabolism	Ly et al., 2014
Mouse (PIGF knockout model)	LFQ	↑ VIM, HIST1HE, TUBB6, CKB, SNAP25, GNAO1, ATPO, GNB2 ↓ MAP2, PRDX6, SLC6A11, CCT8, HNMPAB, MECP2	≥2 or ≤0.5	*P* ≤ 0.05	G-protein activation, protein folding, mRNA splicing, synapse transmission, insulin secretion	Saddala et al., 2018
Human donors (diabetic retinas with/without glial activation)		↑ NDUFA9, APOE, ACO2, ATP5L, SOD2, ACTB, DNM3 ↓ TUBA1B, SDHB, MAPK1, SDHA, PGK1, ENO1, GPI	≥2 or ≤0.5	*P* ≤ 0.05	Unfolded protein response, dopamine degradation, Parkinson’s signaling, neuregulin signaling, synaptic long term potentiation, amyloid processing	Sundstrom et al., 2018
Mouse (intravitreal NMDA-induced glutamate excitotoxicity model)	LFQ	↑ NDUFA12, RPS10, FKBP1A, SBP1, SAE1, FKBP4, DDX28, TCTP, MTA2, RPL35A ↓ MPP2, ERGIC53, TNPO1, FH, AKR1B1, HSPD1, SFXN5, HK1, RAB18, MIC60	≥1.5 or ≤0.67	*P* ≤ 0.05	Neutrophil degranulation, MHC class II antigen presentation, ATP Synthesis, TCA cycle, RHOA signaling, estrogen receptor signaling	Starr et al., 2024

This table compiles key dysregulated proteins published in the past ten years using human donor tissues or animal models. Each entry includes the study, source of tissue, mass spectrometry method, major upregulated (↑) and downregulated proteins (↓) proteins, fold change, *P*-values or adjusted *P*-values (*Q*-values) and implicated biological pathways. ATP: Adenosine triphosphate; DEPs: differentially expressed proteins; FC: fold change; LFQ: label-free quantitative; NMDA: N-methyl-D-aspartate; TCA: tricarboxylic acid.

## Retinitis Pigmentosa

Retinitis pigmentosa (RP) is considered a genetically heterogeneous group of inherited retinal neurodegenerative diseases caused by mutations, affecting approximately 1.5 million people worldwide with an estimated prevalence of around 1 in every 5000 worldwide (Sorrentino et al., 2016). Also known as hereditary retinal dystrophy, this condition progresses through stages that include night blindness, peripheral vision loss, and ultimately results in complete blindness. The symptoms are usually bilaterally, but sometimes there are reports of unilateral eye involvements (Dias et al., 2018). Around 116 genes have been linked to RP inheritance via autosomal dominant, autosomal recessive, X-linked, or sporadic (Daiger et al., 2013). In non-syndromic RP, these genes are involved in biological functions such as phototransduction pathway, transcription, ciliary movements, and visual cycle. Despite being a heterogenous genetic disorder, RP clinically leads to retinal dysfunction and photoreceptor degeneration (Han et al., 2013). Most mutations affect rod and cone photoreceptors, which constitute up to 80% of the retina (Murenu et al., 2021).

Notwithstanding the advances in genetic characterization, therapeutic options have remained limited. Current treatment involves vitamin A supplementation, which has controversial efficacy and potential adverse effects, and newer therapies such as gene therapy are only available for select cases with mutation in their RPE65 gene (Liu et al., 2024a). Thus, understanding photoreceptor degeneration mechanisms is essential to develop mutation-independent therapies, as delaying photoreceptor loss could significantly improve the quality of life for RP patients.

In an early proteomics study on RP, retinal dysplasia and degeneration chicken retina were analyzed using 2-DE proteomics as an animal model resembling severe human retinitis pigmentosa. Several differentially expressed spots were identified by matrix-assisted laser desorption/ionization time-of-flight mass spectrometry MS, including Scn1, which exhibited the most significant variation and other proteins involved in neuroprotection, transcription, and microtubule dynamics (Finnegan et al., 2010). Later three shotgun studies investigated the animal models of RP. In the first study, Ly et al. (2016) explored the neurodegenerative mechanisms in the *rd10* mouse, a RP model with delayed and milder retinal degeneration compared to rd1. Using a modified high-recovery filter-aided sample preparation approach, coupled with label-free quantitative MS, nearly 3000 proteins were profiled across three degenerative stages. Peak degeneration revealed 57 differentially DEPs, including downregulated photoreceptor proteins and upregulated signaling proteins such as glial fibrillary acidic protein, STAT3, and Stat1 (Ly et al., 2016).

In the second study, Wert et al. (2020) performed a proteomic analysis of retinal tissues and liquid vitreous from autosomal recessive RP patients and mice with mutation in *PDE6A*. Based on the results of proteomic analysis, pathways such as fatty-acid synthesis, oxidative phosphorylation, and the tricarboxylic acid cycle were selected for intervention. They found that targeting these pathways with a ketogenic diet and ɑ-ketoglutarate provided neuroprotection, increased docosahexaenoic acid levels, and improved retinal function. This finding is notable as it demonstrates that restoration of metabolites that correlated with proteomic alterations, such as tricarboxylic acid cycle, prolongs retinal function and protects photoreceptors in RP pathology (Wert et al., 2020). The last label free study analyzed the *rd10* mouse model and identified approximately 3700 proteins at different degenerative stages. Bioinformatic analysis of DEPs (222 at 5 weeks, 289 at 8 weeks) revealed disruptions in phototransduction, cilium assembly, and protein localization pathways, with 25 RP-associated proteins directly interacting with PDE6B protein. These findings, validated by quantitative polymerase chain reaction, western blotting, and immunohistochemistry, provide insights into RP pathogenesis and the function of this protein (Yang et al., 2024b).

In a recent iTRAQ LC-MS/MS proteomics study, the retinas of *rd10* mouse model were analyzed, resulting in the quantification of 42 proteins as differentially expressed after MyD88 inhibition. This study suggests that inhibiting MyD88 in mouse models enhanced photoreceptor survival, potentially through altered cytokine levels and neuroprotective microglia. Notably, increased crystallins and stress-response chaperones were associated with enhanced anti-apoptotic and neuroprotective pathways. These findings suggest MyD88 inhibition as a potential strategy for modulating inflammation to protect photoreceptors in RP animal model (Carmy‐Bennun et al., 2021). Recent research in this field has shifted toward proteomics-based biomarker discovery and finding new therapeutic targets using eye or body fluids (e.g., aqueous humor, plasma, serum, and tear), as highlighted in several reviews (García-Quintanilla et al., 2022; Beutgen and Graumann, 2024; Wolf et al., 2024). However, in this review, we stayed focused on retinal proteomics and did not include current literature on biofluids.

**Table 5** provides an overview of key DEPs reported in recent proteomics studies of RP, focusing on findings from commonly used mouse models. These investigations, employing both LFQ and iTRAQ mass spectrometry techniques, consistently revealed an increased abundance of glial and stress-associated proteins, such as glial fibrillary acidic protein, indicative of pronounced gliosis and inflammatory processes in degenerating retinae. In parallel, multiple studies highlighted upregulation of mitochondrial and metabolic proteins (e.g., NDUFS8, GAPDH, SDHB, and ALDH1A1), underscoring metabolic adaptation and enhanced oxidative pathways as photoreceptors deteriorate. Overall, these proteomic studies converge on themes of mitochondrial stress, impaired photoreceptor biology, and reactive gliosis, collectively shaping the molecular landscape of RP progression.

**Table 5:** Summary of DEPs identified in proteomics studies on retinitis pigmentosa

Source	Method	Key DEPs	FC	*P/Q*	Pathways	Reference
Mouse (rd10 mutant model)	LFQ	↑ MVP, STAT1, STAT3, GFAP, VIM, VCAM1, GBP1, MVP ↓ SLC24A1, PDE6B, CADM1, NDUFB4, NNT, ROM1, GRK1	≥ 1.3 or ≤ 0.77	*Q* ≤ 0.05	Signal transduction, phototransduction processes, protein binding, rod photoreceptor function	Ly et al., 2016
Mouse (arRP mice with Pde6α mutation)	LFQ	↑ NDUFS8, GAPDH, SDHB, ALDH1A1, PGAM1, SLC25A4, LAMB1, VDAC2 ↓ DBN1, ACTG1, PVALB, FLNA, SPTBN1, STMN2, RHO, DPYSL2, MAP2, ATP1A2	≥ 2 or ≤ 0.5	*P* ≤ 0.05	Oxidative pathways, TCA cycle, pyrimidine metabolism, protein turnover, phototransduction, oxidative phosphorylation	Wert et al., 2020
Mouse (rd10 mutant model)	iTRAQ	↑ PPP1CB, CRYBA1, RPS7, HP1BP3, ADSS, HNRNPU, LGALSL ↓ ASNA1, NCL, RPS9, NPEPPS, BSG, SLC1A3, MPP2, TUBB4A, DCPS, GARS	≥ 1.2 or ≤ 0.83	*P* ≤ 0.05	Eye development, peptide metabolic process, response to hypoxia, protein binding, tubulin complex assembly, pyrophosphatase activity	Carmy–Bennun et al., 2021
Mouse (rd10 mutant model)	LFQ	↑ APOE, GFAP, VIM, ASS1, MGST1, PRKCB, SEPTIN2, LAMB2, EXOC8 ↓ RHO, PRPH2, GNB1, ROM1, BBS1, ARL6, CROCC, HK2	≥ 2 or ≤ 0.5	*P* ≤ 0.05	Visual perception, phototransduction, response to stimulus, protein localization, cilium assembly	Yang et al., 2024b

This table compiles key dysregulated proteins published in the past ten years using human donor tissues or animal models. Each entry includes the study, source of tissue, mass spectrometry method, major upregulated (↑) and downregulated proteins (↓) proteins, fold change, *P*-values or adjusted *P*-values (*Q*-values) and implicated biological pathways. arRP: Autosomal recessive retinitis pigmentosa; DEPs: differentially expressed proteins; FC: fold change; iTRAQ: isobaric tags for relative and absolute quantification; LFQ: label-free quantitative; TCA: tricarboxylic acid.

## Comparative Proteomics of the Retina and Brain in Alzheimer’s Disease

AD is a progressive neurodegenerative disorder characterized by a gradual and irreversible decline in cognitive and functional abilities, with limited disease-modifying treatments currently available (Scheltens et al., 2016; Dhillon, 2021; Haddad et al., 2022; Lista et al., 2022; McDade et al., 2022; Boxer and Sperling, 2023; Sims et al., 2023; van Dyck et al., 2023). AD progresses along a clinical-biological continuum, featuring extended asymptomatic stages driven by underlying pathological processes, particularly Aβ aggregation, which can begin more than 20 years before clinical symptoms appear (Holtzman et al., 2011; Sperling et al., 2011, 2013; Bateman et al., 2012; Buchhave et al., 2012; Fagan et al., 2014; Dubois et al., 2016; Hampel et al., 2021a). Despite advances, early detection of AD pathology remains challenging in clinical practice (Porsteinsson et al., 2021; Hu et al., 2023). However, the lengthy preclinical phase offers a critical window for early diagnosis and intervention, potentially preventing significant structural and functional brain damage (Hampel et al., 2019).

AD is characterized by the accumulation of brain Aβ plaques along with neurofibrillary tangles comprised of hyperphosphorylated tau (Masters et al., 1985; Grundke-Iqbal et al., 1986; Kosik et al., 1986; De-Paula et al., 2012; Hampel et al., 2021b; Yarns et al., 2022). In addition, chronic neuroinflammation, including brain-resident glial activation and peripheral immune cell infiltration, along with blood-brain barrier dysfunctions, characterize the pathophysiological processes of AD (Montagne et al., 2017; Zhong et al., 2018; Ni Chasaide and Lynch, 2020; Welikovitch et al., 2020; Pascoal et al., 2021). These pathological changes ultimately lead to neurodegeneration and synaptic dysfunction, culminating in cognitive deficits and, in the later stages of the disease, severe dementia, disability, and ultimately death.

Similar pattern of neurodegeneration, where axonal damage precedes cell death, is seen in glaucoma, AD, Parkinson’s disease (PD), and amyotrophic lateral sclerosis, suggesting shared pathological mechanisms between these neurodegenerative conditions (Selkoe, 2002; Jakobs et al., 2005; Fischer and Glass, 2007). Recent studies in primates and humans have revealed that glaucomatous damage extends beyond the eye and optic nerve into the central visual pathway. This trans-synaptic degeneration, similar to that observed in AD and PD, reinforces the notion of glaucoma as a neurodegenerative disease of the CNS (Gupta and Yücel, 2007; Yücel and Gupta, 2008). Furthermore, AD hallmarks implicated in neuronal death and disease progression, such as Aβ (Goldblum et al., 2007; Yin et al., 2008) and hyperphosphorylated tau (Yoneda et al., 2005; Gupta et al., 2008), have been detected in glaucoma.

Notably, the retina shares common pathological features with the brain in AD (Gaire et al., 2024), classifying AD as an ocular neurodegenerative disease. Indeed, emerging evidence from human histopathological, biochemical, and *in vivo* imaging studies has revealed that AD-related pathological changes not only affect the brain but also manifest in the retina of patients (Koronyo-Hamaoui et al., 2011; Koronyo et al., 2017, 2023; Shi et al., 2020; Hart de Ruyter et al., 2023; Shi et al., 2023). The pathological hallmarks identified in the retina of AD patients were accompanied by vascular abnormalities, gliosis, and neuronal degeneration (Hinton et al., 1986; Blanks et al., 1989; Sadun and Bassi, 1990; Berisha et al., 2007; Grimaldi et al., 2019; Xu et al., 2022). Retinal Aβ deposition and other AD-related pathology seemed to follow a similar trajectory to that of the brain at different disease stages (Gupta et al., 2016; Koronyo et al., 2017, 2023; Doustar et al., 2020; Schultz et al., 2020; Shi et al., 2020, 2023; Habiba et al., 2021).

**Figure 2:**
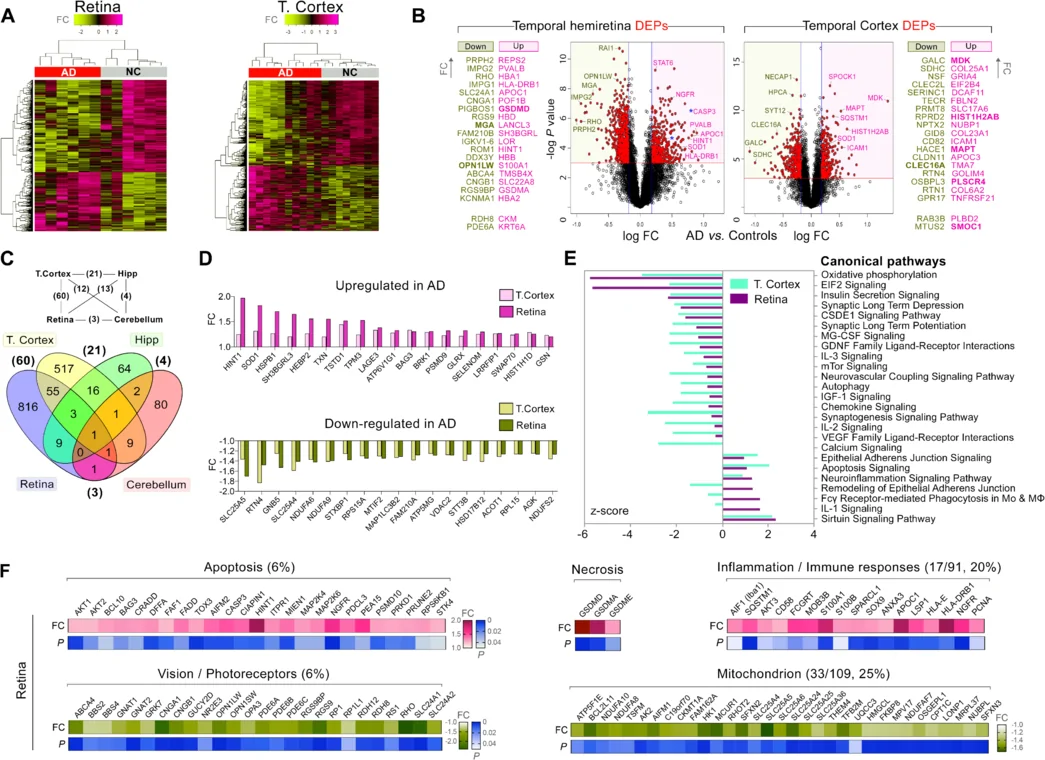
Proteome profiling of AD retina and brain. (A) Heatmaps display the proteomics profiling with detectable protein hierarchies as identified by mass spectrometry analysis on protein homogenates from temporal hemiretinas (*n* = 6 AD, *n* = 6 NC) and temporal cortices (*n* = 10 AD, *n* = 8 NC) of AD patients versus individuals with NC controls. The DEPs and FC are presented for upregulated (pink) and downregulated (green) DEPs. (B) Volcano plots of top 20 up- or downregulated DEPs are organized by FC (lowest *P* values highlighted in bold) in retinas and temporal cortices from AD versus NC subjects (DEPs marked by red circles). (C) Venn diagram depicting the number of overlapping DEPs according to statistical significance (*P* < 0.05) and 1.2-FC threshold criteria in the 4 analyzed CNS tissues; the number of common DEPs between paired CNS tissues (bold). (D) Common retinal and cortical upregulated and downregulated DEPs in AD patients versus NC controls. (E) Canonical pathway analysis of top upregulated and downregulated biological functions in AD versus NC retinas based on Z-scores. (F) DAVID biological classification analysis displays major upregulated and downregulated pathways, presenting the top upregulated DEPs (pink) related to apoptosis, necrosis, and inflammation/immune responses, as well as the top downregulated DEPs (green) associated with photoreceptor degeneration and mitochondrial dysfunction in AD versus NC retinas. Lower blue bars represent the magnitude of *P* values. Percentages indicate the fraction of each category of total upregulated or downregulated DEPs. Reprinted with permission from Koronyo et al. (2023). AD: Alzheimer’s disease; CNS: central nervous system; DAVID: database for annotation, visualization and integrated discovery; DEPs: differentially expressed proteins; FC: fold change; NC: normal cognition; T.: temporal.

A recent study by Koronyo et al., (2023) analyzed the proteome signatures of AD retinas and brains, revealing activation of specific inflammatory and neurodegenerative processes and inhibition of oxidative phosphorylation and mitochondrial pathways (**Figure 2**). Proteins were isolated from the temporal hemiretina and 3 different brain regions (temporal cortex, hippocampus, and cerebellum) of AD patients and age- and sex-matched individuals with normal cognition. Applying the LC–MS/MS with a Q Exactive Orbitrap, this study identified 8286 retinal and 7312 brain targets. Among these, 886 DEPs were detected in retain, 602 in the temporal cortex, 96 in the hippocampus, and 95 in the cerebellum of AD patients versus normal cognition controls (Koronyo et al., 2023). The authors reported common profiles in the temporal retina and cerebral temporal cortex, as shown by similar heatmap patterns and volcano plots (**Figure 2A** and **B**). As compared with other brain regions, the proteome profiles of the temporal cortex were the most overlapping with the retina in the AD patients (**Figure 2C**). Overall, 60 AD-related overlapping DEPs were identified between the retina and temporal cortex, whereas there were only 13 common DEPs between the retina and hippocampus and 3 between the retina and cerebellum (**Figure 2C**), suggesting that AD pathological changes in the temporal retina are more closely associated with cortical changes.

Among the top 10 upregulated DEPs shared between the temporal cortex and retina of AD patients (**Figure 2D**), various proteins are implicated in disease pathogenesis and tissue injury responses (Koronyo et al., 2023). The HEBP2 (Heme-binding protein 2) promotes mitochondrial permeability transition and necrotic cell death, which are integral processes of mitochondrial dysfunction and neurotoxicity in AD (Pérez et al., 2018). Similarly, the thiosulfate sulfurtransferase-like domain-containing protein 1 (TSTD1) modulates mitochondrial and lipid metabolism, which are increasingly recognized as critical components of AD-related neurodegeneration (Zheng et al., 2021). Multiple other mitochondria-related proteins were also similarly downregulated in the AD brain and retina, some of which are discussed below, implicating a severe mitochondrial dysfunction shared between the AD retina and brain. The superoxide dismutase 1 (SOD1) activity and expression are elevated in fibroblast cell lines derived from familial AD patients (Zemlan et al., 1989; Norambuena et al., 2022). Notably, proteomics data indicate that SOD1 is significantly upregulated in the AD retina and brain. As a key antioxidant enzyme, SOD1 mitigates reactive oxygen species-mediated damage; however, its increased expression in AD likely reflects a compensatory response to oxidative stress. Notably, aberrant SOD1 activity or aggregation has been linked to neurodegeneration and disease progression (Choi et al., 2005). Likewise, thioredoxin that is increased in the AD retina and temporal cortex, has been associated with modulation of oxidative stress, inflammation, and pyroptotic cell death and was reported to be elevated in the cerebrospinal fluid and plasma of AD patients (Cornelius et al., 2013; Arodin et al., 2014; Jia et al., 2025). Furthermore, the heat shock protein family B member 1 (HSPB1) confers cellular protection under stress conditions such as inflammation and toxin exposure. In AD, HSPB1 is often upregulated, particularly in reactive astrocytes surrounding Aβ plaques. This HSPB1 upregulation is believed to facilitate sequestering of toxic Aβ oligomers and related harmful effects on neurons (Ojha et al., 2011; Yang et al., 2024a); however, the role of HSPB1 in AD pathogenesis remains largely unknown. Overall, these stress-related proteins (SOD1, thioredoxin, and HSPB1) are elevated in both the brain and retina of AD patients and likely reflect compensatory mechanisms activated in response to chronic oxidative stress and cellular injury.

Another upregulated protein in the AD retina and temporal cortex is the histidine triad nucleotide-binding protein 1 (HINT1), a purine nucleotide hydrolase, which has been involved in neuroplasticity regulation and neuronal pro-apoptotic function (Liu et al., 2017; Kang et al., 2025). While its role in AD pathogenesis remains undefined, its known functions suggest a potential contribution to dysregulated proteostasis and neurodegenerative mechanisms. In addition, the tropomyosin 3 (TPM3) that plays a fundamental role in actin cytoskeleton organization is upregulated in the AD retina and brain. TPM3 expression was shown to be elevated in the white matter and hippocampus of AD patients compared to controls, potentially contributing to disrupted neuronal function through abnormal cytoskeletal organization (Owen et al., 2009; Castaño et al., 2013). Further upregulated protein in the AD retina and brain is the glutaredoxin-1. Glutaredoxin-1 is a thiol-disulfide oxidoreductase that plays a key role in maintaining cellular redox homeostasis and its levels were elevated in the brains of AD patients. Moreover, in SH-SY5Y neuronal cells, glutaredoxin-1 undergoes oxidation in response to Aβ exposure, a modification that may activate signaling pathways contributing to neurotoxicity (Akterin et al., 2006). Two other shared retina and brain upregulated proteins are related to inflammation. The SH3 domain-binding glutamic acid-rich-like protein 3 (SH3BGRL3) has been associated with resistance to tumor necrosis factor-α-induced apoptosis (Xu et al., 2005) and its elevated levels in the cerebrospinal fluid have been proposed as a potential biomarker for early-onset AD (Li et al., 2024). Notably, the lymphocyte activation gene 3 (LAGE3; also known as LAG3), which is expressed by neurons and activated microglia and implicated in neuroinflammation, is found to be significantly upregulated in the hippocampus, temporal cortex, and retina of AD patients (Koronyo et al., 2023). Previous studies demonstrated that LAG3 expression is increased in microglia upon activation by interferon-gamma, and this expression is regulated by the STAT1 pathway (Morisaki et al., 2023). Further, LAG3 has been shown to bind specifically to fibrillar tau, suggesting its involvement in the propagation of tau pathology in AD (Chen et al., 2024).

The top 10 significantly downregulated DEPs identified in both the AD retina and temporal cortex (**Figure 2D**) are involved in mitochondrial function, synaptic integrity, and neurodegeneration (Koronyo et al., 2023). The solute carrier family 25 member 5 (SLC25A5) plays a critical role in maintaining mitochondrial membrane potential, regulating calcium homeostasis, and inhibiting apoptosis (Tian et al., 2024; Qian et al., 2025). Its downregulation in the AD brain and retina likely contributes to AD pathology by disrupting these essential mitochondrial functions and calcium signaling. Similarly, another solute carrier protein SLC25A4 (also known as adenine nucleotide translocator 1), which mediates adenosine diphosphate/ATP exchange across the mitochondrial inner membrane, is downregulated in both AD brains and retinas. Its dysregulation in AD brain has been linked to impaired mitochondrial bioenergetics and tauopathy-associated pathology (Tracy et al., 2022; Tian et al., 2024). Accordingly, the NADH: ubiquinone oxidoreductase subunit A6 (NDUFA6), a core component of mitochondrial complex I, is implicated in mitochondrial dysfunction and has been identified as a genetic risk factor for AD (Cheng et al., 2022). Carriers of the NDUFAF6 rs6982393 variant exhibit increased AD susceptibility (Cheng et al., 2022). Similarly, the downregulation of NDUFA9 further suggests a disruption in complex I activity, contributing to mitochondrial dysfunction and neurodegenerative processes in AD (Adav et al., 2019). In addition, reduced mitochondrial translation initiation factor 2 (MTIF2), a critical regulator of mitochondrial protein synthesis and network maintenance (Overman et al., 2003), potentially contributes to mitochondrial failure and subsequent neurodegeneration in the AD retina and brain.

In relation to the amyloidogenic pathway, proteome expression profiles in the AD brain and retina indicate downregulation of the reticulon 4 (also known as Nogo) that interacts with BACE1 to suppress Aβ production (Kume et al., 2009). Elevated reticulon 4 expression has been associated with reduced Aβ secretion and improved cognitive performance in AD mouse models (Murayama et al., 2006; Masliah et al., 2010), suggesting that its downregulation in the AD brain and retina may contribute to Aβ accumulation. Notably, the G protein subunit beta 5 expression has been reduced in brains from AD patients and animal models. Cerebral G protein subunit beta 5 reduction correlated with increased Aβ plaque burden and neurofibrillary tangle formation (Zhang et al., 2024). Moreover, G protein subunit beta 5 has been proposed as a potential AD risk gene, with Gnb5 variant heterozygosity exacerbating AD-related neuropathology in transgenic mice (Zhang et al., 2024; Chen et al., 2025). Therefore, these downregulated proteins may have contributed to increased cerebral and retinal Aβ and tau pathology in AD patients.

Another downregulated protein in the AD brain and retina includes the syntaxin binding protein 1, which is essential for synaptic vesicle exocytosis by regulating syntaxin-mediated neurotransmitter release (Yuan et al., 2023). Its reduced cerebral and retinal expression in AD patients suggests impaired synaptic function. Furthermore, the ribosomal protein S15A has been detected in AD-associated microglia and cerebral capillaries, suggesting a role in neuroinflammation and blood-brain barrier dysfunction (Patel et al., 2020; Suzuki et al., 2022). Finally, the microtubule-associated protein 1 light chain 3 beta 2 (MAP1LC3B2) is involved in autophagosome formation, a key step in autophagy. Dysfunctional cerebral autophagy in AD was reported to lead to the accumulation of misfolded proteins such as Aβ and tau, promoting neurodegeneration (Shpilka et al., 2011; Zhang et al., 2021). Hence, downregulation of MAP1LC3B2, as identified by mass spectrometry analysis, can be involved in Aβ and tau accumulation in both the retina and temporal cortex of AD patients. Collectively, these shared dysregulated proteins in the AD brain and retina highlight mitochondrial impairment as a central feature of AD pathology, potentially contributing to oxidative stress response, neuroinflammation, and microglial activation, as well as progressive neurodegenerative processes in both the brain and retina (**Figure 2E**).

In the AD retina, among the top 20 upregulated DEPs many proteins were associated with immune activation and cell death, including MHC class II molecules (HLA-DRB1), HLA-E, apolipoprotein C-I (APOC1), AIF1 (IBA-1), CD68, S100β, caspase-3, gasdermin-D (GSDMD), and GSDME. GSDMD, a key effector of pyroptosis was notably elevated. APOC1, predominantly expressed by disease-associated microglia, modulates innate immune responses and cholesterol efflux. In the CNS, APOC1 has been linked to neuroinflammation and AD pathology (cortical atrophy and cognitive deficits) and is upregulated in AD brains and in individuals carrying the ApoE4 allele (Zhou et al., 2014a, b; Kulminski et al., 2022; Oh et al., 2025). Indeed, similar to the AD brains, approximately 20% of upregulated DEPs in the AD retinas were related to immune responses, involving lymphocyte and myelomonocyte activation in the presence of neuroinflammation and infection.

Among the top 20 most substantially downregulated DEPs in the AD retina, over 70% were photoreceptor-specific markers—retinal neurons whose nuclei reside within the ONL, a region that harbors prominent Aβ accumulation in AD dementia patients (Koronyo et al., 2023). These downregulated proteins include peripherin-2 and interphotoreceptor matrix proteoglycans 1 and 2, which are expressed by both rod and cone photoreceptors, as well as the rod-specific visual pigment rhodopsin (also known as opsin-2 or OPN2) and the cone-specific opsin OPN1LW, a long-wavelength-sensitive photopigment. Notably, approximately 25% of the downregulated DEPs were associated with mitochondrial function, consistent with well-documented mitochondrial impairment in AD brains. These proteomic findings indicate profound photoreceptor degeneration accompanied by mitochondrial dysfunction in the temporal hemiretina of individuals with AD dementia, with potential implications for deficits in color discrimination and contrast sensitivity observed in these patients.

As compared to the control retina of normal cognition, the DAVID (database for annotation, visualization and integrated discovery) functional classification exhibited that apoptosis, necrosis, and inflammation were the most activated, while vision/photoreceptors, oxidative phosphorylation/mitochondria, and transcription/translation were the most inhibited pathways in AD retina (**Figure 2F**). The PANTHER (protein annotation through evolutionary relationship) protein classification revealed similar patterns for AD retinas and temporal cortices, in contrast to hippocampi and cerebella. Ingenuity pathway analysis in AD versus normal cognition retinas indicated that the top activated biological functions were related to retinal/photoreceptor degeneration and morbidity or mortality, while the top inhibited functions were related to the quantity of retinal cells and photoreceptors. By integrating advanced bioinformatic tools such as Cytoscape’s StringApp, DAVID, PANTHER, and ingenuity pathway analysis, Koronyo et al. (2023) classified and visualized differentially expressed proteins within AD retinas. Results showcased heightened inflammatory, apoptotic, and necrotic pathways, along with suppressed oxidative phosphorylation and photoreceptor function (Koronyo et al., 2023). Collectively, these results underscore the importance of retinal proteomics for uncovering neurodegenerative mechanisms and highlight the potential of the retina as an accessible biomarker for AD diagnosis.

A few studies in animal models also highlighted the retinal proteome signatures in AD. Iqbal et al. (2019) described the proteomic alterations in the retinas of 2-, 4-, and 6-month-old 3×Tg-AD mice using iTRAQ proteomics technology. Among the total identified proteins, 121 proteins (71 up-regulated and 50 down-regulated), 79 proteins (51 up-regulated and 28 down-regulated), and 153 proteins (37 up-regulated and 116 down-regulated) were found to be significantly differentially expressed in the retinas of 2-, 4-, and 6-month-old mice, respectively. Seventeen DEPs were common across all three age groups. Bioinformatics analysis of these DEPs highlighted their involvement in critical AD-related biological processes, including metabolic pathways, structural cascades, retinal processes, synaptic and neuronal proteins, and eye lens-associated phenomena (Iqbal et al., 2019).

El-Darzi et al. (2022) used proteomics analysis to identify biological processes enriched with differentially expressed genes and proteins associated with retinal vascular lesions in 5×FAD mice. They identified a total of 2644 proteins, with 30 proteins downregulated and 43 proteins upregulated. The highest number of DEPs (9) were involved in the regulation of cell adhesion. Other identified DEPs were associated with various biological processes, including neuron development, regulation of vesicle-mediated transport, translation, regulation of phagocytosis, RNA localization, regulation of the proteasomal ubiquitin-dependent protein catabolic process, and protein dephosphorylation. These findings provide insights into the molecular changes linked to retinal vascular lesions in AD. Similarly, Mirzaei et al. (2019) used a multiplexed proteomics approach with isobaric tandem mass tags, followed by functional and protein-protein interaction analyses, to study the molecular effects of Aβ accumulation and AD progression in the retinas of 2.5-month-old and 8-month-old APP/PS1 mice. They identified approximately 2000 proteins in both younger and older APP/PS1 mice retinas. In the younger mice, 50 proteins were upregulated and 36 were downregulated, while in the older mice, 85 proteins were upregulated and 79 were downregulated. Amyloid precursor protein (APP) was consistently upregulated two- to threefold in both age groups. In the older APP/PS1 mice, elevated levels of proteolytic enzymes cathepsin D, presenilin 2, and nicastrin, associated with APP processing, were observed. Additionally, increased levels of proteasomal proteins Psma5, Psmd3, and Psmb2 were found in the older AD retinas. In contrast to the younger animals, the retinas of older mice exhibited significant downregulation of proteins involved in protein synthesis and elongation, such as Eef1a1, Rpl35a, Mrpl2, and Eef1e1 (Mirzaei et al., 2019).

Doustar et al. (2020) used SWATH-MS analyses on retinal and brain tissues to identify parallel proteome changes between these two tissues in AD transgenic mice. They found significant upregulation of several inflammation-related proteins in both retinal and cerebral tissues of old AD transgenic mice, including intercellular adhesion molecule 1, glutamine synthetase, and histocompatibility antigen 11. Additionally, they observed common proteins involved in amyloid processing and clearance in both the brain and retina. These proteins included Aβ, clusterin, and lysosomal-associated membrane proteins 1 and 2. These findings highlight the similar molecular changes occurring in both the brain and retina in the context of AD.

To identify the molecular mechanisms by which knockdown of microRNA-155 in microglia affects the wild-type and AD model retina, Shi et al. (2022) conducted a global protein expression analysis using mass spectrometry. They found commonly upregulated protein levels of METTL3, METTL14, and METTL16. The largest changes were observed in proteins related to metabolite interconversion enzymes and translational proteins. Other identified proteins were associated with phosphoinositide 3-kinase-Akt related pathways, including insulin secretion, mammalian target of rapamycin, eukaryotic initiation factor 2, and sirtuin signaling pathways. Additionally, retinal proteins Spp1 and Cxcl8 were identified. These findings provide insights into the molecular effects of microRNA-155 knockdown in microglia on retinal health in both wild-type and AD models.

Using nano-LC-MS/MS analysis, Fridlich et al. (2009) identified 90 proteins that interact with the Rod-derived cone viability factor (RdCVFL) in mice. Among these, some proteins were associated with apoptosis, such as apoptosis inhibitor 5 and poly(adenosine diphosphate-ribose) polymerase 1, while others were linked to neurodegenerative diseases, including tau protein (Mapt). The analysis of the RdCVFL interactome revealed three main clusters of proteins: ribosomal proteins, spliceosome proteins, and actin-binding proteins. Within the cluster of actin-binding proteins, tau protein was identified, which is known to be involved in neurodegenerative diseases such as AD. Notably, the level of tau phosphorylation was increased in the retinas of Nxnl1^−/−^ mice (Fridlich et al., 2009).

Deng et al. (2019) studied the time- and dose-dependent effects of Aβ in 661W photoreceptor cells using a TMT labeling-based quantitative approach. They found that Aβ treatment led to increased tau phosphorylation, glycogen synthase kinase-3 beta dysregulation, and reduced cell viability in a dose- and time-dependent manner. Interestingly, proteins involved in ribosomal machinery homeostasis, mitochondrial function, and cytoskeletal organization were affected in the early stages of Aβ exposure, indicating key insights into the specific molecular changes in photoreceptor during AD pathologies. Integral Membrane Protein 2B (ITM2B) is a type II transmembrane protein associated with Alzheimer-like autosomal dominant disorders, characterized by early-onset progressive dementia and cerebellar ataxia. Wohlschlegel et al. (2021) performed quantitative proteomics on the ITM2B interactome in the human retina. They identified 457 partners of ITM2B, with 8 of these proteins involved in visual transduction. Other identified proteins were involved in various biological functions, including microtubule organization, protein translation, and notably, mitochondrial homeostasis.

Mirshahvaladi et al. (2024) employed a multiplex quantitative proteomics approach using TMT technology, followed by functional enrichment and protein-protein interaction analysis to identify CNS proteome changes in neuroserpin-deficient (NS^−/−^) mice compared to control mice. They detected around 5000 proteins in each tissue, including the retina, optic nerve, frontal cortex, visual cortex, and cerebellum, resulting in a pool of more than 1200 DEPs. Principal component analysis and hierarchical clustering highlighted similarities and differences between the retina and various brain regions and distinguished NS^−/−^ proteome signatures from control samples. Functional pathway analysis revealed region-specific changes in each region that can potentially explain how Serpini1 acts as a neuroprotective molecule in the CNS (Mirshahvaladi et al., 2024).

### Alteration of post-translational modifications in neurodegenerative retinal diseases

While high throughput proteomics studies have explored protein expression changes in various animal models and clinical samples of retinal neurodegenerative diseases, the PTM profile of the retina in these conditions remains largely unknown. Nonetheless, in “Liquid Biopsy Proteomics in Ophthalmology” (Wolf et al., 2024), the authors demonstrate how advanced mass spectrometry-based analyses of minimally invasive liquid biopsies can overcome the limited availability of ocular tissues for biomarker discovery. Through proteomic profiling of biological fluids, combined with machine learning approaches to decipher the resulting high-dimensional datasets, disease-specific protein signatures are identified for more accurate diagnosis, prognosis, and assessment of therapeutic outcomes. Critically, the use of artificial intelligence-driven computational pipelines incorporating feature extraction, dimensionality reduction, and predictive modeling refines stratification of pathologies such as age-related macular degeneration and diabetic retinopathy and can similarly be extended to neurodegenerative disorders manifesting in the retina. Notably, the authors emphasize that integrating repeated cross-validation and robust classification strategies strengthens disease-specific biomarker discovery, enabling the identification of low-abundance proteins and subtle proteomic changes indicative of disease onset or progression.

The fluid-based strategy, complemented by integration with other omics approaches, facilitates earlier detection of altered proteomes in the eye. In the context of neurodegenerative diseases, repeated sampling of these ocular fluids permits ongoing monitoring of disease progression, ultimately aiding in the identification of novel biomarker panels and providing a personalized, artificial intelligence-guided approach to therapeutic interventions. The team also demonstrated that PTMs are covalent, often enzymatic modification of proteins that occur after translation (Thygesen et al., 2018), adding extra adjusting mechanisms to regulate cellular and signaling pathways (Gajadhar and White, 2014). PTMs can occur on amino acid side chains or protein termini, either modifying existing functional groups or adding new groups. There are over 400 characterized PTMs, which can involve the addition of functional groups (phosphorylation/acetylation), polypeptides (SUMOylation/ubiquitination), or complex molecules (glycosylation/lipidation) (Gupta et al., 2021a). These protein modifications can induce allosteric structural changes, activate or inhibit enzymes, alter protein localization within cells and modulate protein-protein interactions (Johnson, 2009). Recent advancements in proteome studies have enabled the analysis of retinal neurodegenerative diseases, the study of post-translational modifications in the diseased retina remains in its early stages. In this section, we review major post-translational modification findings on retinal tissues in four retinal diseases: glaucoma, AMD, DR, and RP.

There are many studies focused on individual proteins and their PTM modifications in animal models of glaucoma, but only two high-throughput studies have explored phosphoproteome of mouse retina lysates following optic neuropathies (Lukas et al., 2009; Liu et al., 2020). In one of the most comprehensive studies, Luu et al. (2023) conducted an integrating single-cell transcriptomics, proteomics, and phosphoproteomics to identify universal molecular mechanisms underlying impaired physiological stress resilience in various age-related and inherited retinal degeneration. In addition to *in vitro* models, they used various murine models, including photosensitive double-knockout mouse (Abca4^−/−^ /Rdh8^−/−^) exhibiting pathological hallmarks of human AMD, streptozotocin model of diabetic retinopathy and *rd10* model of autosomal recessive retinitis pigmentosa (Luu et al., 2023). Their goal was to explore the potential of cyclic nucleotide phosphodiesterases to enhance stress resilience in degenerating retina. Their findings suggested that phosphodiesterases are specific key regulators of intracellular second messengers, activating protective mechanisms while simultaneously inhibiting degenerative processes, thereby having broad applicability across different retinopathies involving acute/chronic stressors.

Our literature review highlights the need for future research on post-translational modifications to accelerate the development of fields that will generate the knowledge necessary to design effective treatments. Given their reversible nature, PTMs represent a promising avenue for the development of innovative therapeutics. Phosphorylation continues to be the most studied type of PTM, possibly due to the availability of high-quality Ser/Thr antibodies and well-established mass spectrometry computational tools. The large-scale acetylation/methylation/glycosylation profiling of the diseased retina remains largely unexplored, offering a promising research direction, particularly on O-GlcNAcylation in the diabetic retina (Yang and Qian, 2017).

### Retinal proteome changes in other neurodegenerative diseases

As observed in AD, retinal proteome changes similarly reflect the brain pathology in other neurological diseases, including PD, MS, and stroke. In PD, α-synuclein, a key protein implicated in neurodegeneration, accumulates not only in the brain but also in the retina of individuals with the disease (Beach et al., 2014; Bodis-Wollner et al., 2014; Ortuño-Lizarán et al., 2018). PD patients also exhibit abnormal retinal thickness, altered electroretinogram responses, and visual impairments (Alves et al., 2023). In addition, significant microvascular dysfunctions have been reported in the PD retina, mirroring similar pathology in the brain (Robbins et al., 2021). Proteomic studies in PD animal models have revealed that DEPs are involved in essential biological pathways, including glycolysis, mitochondrial electron transport, stress response, neuronal survival, and phototransduction. For example, Campello et al. (2013) identified alterations in retinal protein expression patterns that suggest disruptions in energy metabolism, neuroprotective mechanisms, and signal transduction in response to MPTP-induced retinal neurodegeneration in monkey, paralleling mechanisms of neuronal loss seen in both the PD brain and other neurodegenerative diseases. Notably, proteins associated with energy metabolism, stress-related neuroprotection, and visual signal processing were downregulated (Campello et al., 2013).

In MS, extensive studies have documented retinal abnormalities across various disease subtypes, including acute, chronic, relapsing-remitting, and primary or secondary progressive forms. Key retinal changes include significant tissue atrophy, optic disc abnormalities, RNFL and ganglion cell layer thinning, optic nerve degeneration, inflammatory infiltration, and reduced retinal vascular densities compared to healthy controls (Green et al., 2010; Jankowska-Lech et al., 2019; Mrabet et al., 2024; Dulger et al., 2025). In a cuprizone-induced MS-like mouse model, Almuslehi et al. (2022) identified several upregulated and downregulated DEPs in the optic nerve, which were linked to demyelination and glial activation. Similarly, in autoimmune retinopathy patients, Al-Moujahed et al. (2022) reported altered protein expression in the vitreous body. Upregulated proteins included lysozyme C, zinc-alpha-2-glycoprotein, complement factor D, TGF-β–induced protein, β-crystallin B2, and alpha-crystallin A chain. Downregulated proteins included DIP2C, retbindin, and Aβ precursor-like protein 2 (Al-Moujahed et al., 2022).

In stroke, retinal impairment and neurovascular alterations are also commonly observed. These include decreased vessel density, RNFL and ganglion cell layer thinning, increased perimeter acircularity, and reduced foveal avascular zone, all of which signify structural degeneration when compared to healthy individuals (Liu et al., 2021a; Liang et al., 2022). Experimental stroke models in animals have demonstrated microvascular tortuosity, inflammatory responses, retinal degeneration, and optic nerve atrophy (Kumar, 2017). In a rat ischemic model, Stowell et al. showed that ischemic preconditioning induced substantial changes in histone-related protein expression, such as increased levels of histones H2B, H3, and H4, and decreased levels of tri-methylated H3 and mono-ubiquitinated H2A (Stowell et al., 2010).

Collectively, these studies highlight significant retinal abnormalities associated with neurodegenerative diseases. Further investigations are warranted to elucidate functional proteomic alterations in the retina of affected patients and corresponding animal models.

### Future Directions

The field of retinal proteomics in neurodegeneration is rapidly evolving, with several promising directions for future research and clinical applications. The new generation of mass spectrometry platforms, including the latest generation of Orbitrap and time-of-flight instruments, now enable deeper proteome analysis from minute tissue samples (Guo et al., 2025). These developments are particularly relevant for retinal proteomics, where sample quantity is very limited. Another technical issue is high-abundant proteins such as albumin, crystallins, or hemoglobin that mask low-abundance proteins during MS analysis. Depletion of these highly abundant proteins using various chromatography methods can improve the protein coverage (Nakayasu et al., 2021).

Due to the impossibility of obtaining retinal samples from healthy living human eyes, recent focus has shifted towards proteomics analysis of tear/serum, which can be obtained through minimally invasive procedures or even aqueous humor/vitreous humor during surgical procedures (Jay and Gillies, 2012). Unlike traditional tissue biopsies, these liquid samples contain a diverse array of proteins sourced from specialized ocular cells, providing a comprehensive snapshot of tissue heterogeneity and disease mechanisms. To date, comprehensive studies directly comparing retinal tissue and corresponding fluid proteomes are limited, and the extent to which liquid biopsies can serve as true surrogates for retinal molecular changes remains to be fully established.

Extracellular vesicles (EVs) have emerged as important carriers of disease-specific protein signatures in various retinopathies. EVs carry cargo proteins that reflect/contribute to the underlying disease processes and represent their cellular origin, making them ideal biomarker reservoirs for early detection of retinal neurodegenerative events (Shekari et al., 2023). The isolation and proteomic profiling of EVs from vitreous humor, aqueous humor, and tears represents a minimally invasive approach to monitoring retinal health. Recent studies have demonstrated that retinal-derived EVs contain disease-specific protein signatures in AMD (Zhou et al., 2023) and DR (Zhou et al., 2023). Future developments in EV isolation techniques combined with sensitive mass spectrometry will enable longitudinal monitoring of disease progression through liquid biopsy approaches.

Mass spectrometry-based single-cell proteomics (SCP) is emerging as a new approach for analyzing protein expressions, enabling the detection of thousands of proteins and uncovering cellular heterogeneity that remains hidden in traditional bulk analyses (Sanchez-Avila et al., 2025). This trending technology is rapidly gaining traction for investigating complex biological processes and disease mechanisms as SCP allows for precise dissection of proteomic variation between morphologically or transcriptionally similar cells. New technological advances have expanded this tool dramatically, now allowing for the quantification of over 5000 protein groups per cell (Bubis et al., 2025; Ye et al., 2025). Integrating proteomics with other approaches, including transcriptomics, metabolomics and epigenomics has led to the creation of multi-omics cell atlases of human retina and RPE cells in AMD (Senabouth et al., 2022; Liang et al., 2023). These atlases have uncovered changes in gene expression in various cell types, as well as hundreds of quantitative trait loci, which are linked to disease pathology. However, the application of SCP to diseased retina has remains limited. Expanding SCP to pathological contexts offers enormous potential to identify early disease markers, neuroinflammatory processes, and characterize vulnerable cell populations at the molecular level.

Computational tools are increasingly important for extracting meaningful insights from complex proteomic datasets. The TEMPO (Tracing Expression of Multiple Protein Origins) approach enables real-time monitoring of protein profiles in liquid biopsies and can trace proteins to their cellular origins by integrating proteomic data with single-cell RNA sequencing data from various ocular and extraocular tissues, enabling precise identification of protein origins (Wolf et al., 2024). This artificial intelligence-driven approach provides a sophisticated method for age-related protein profiling, leveraging advanced machine learning techniques to extract insights from complex proteomic datasets.

Building on the growing field of retinal proteomics in neurodegenerative diseases, deep-learning tools such as Prosit offer powerful *in silico* methods for biomarker discovery and precision diagnostics by accurately predicting peptide tandem mass spectra, even with limited retinal tissue (Gessulat et al., 2019). Its integration of predicted ion intensities and retention times streamlines high-throughput MS analysis, enhancing peptide-spectrum matching. With collision energy calibration and retina-specific transfer learning, Prosit can detect post-translational modifications or splice variants relevant to diseases such as glaucoma, diabetic retinopathy, and AD. Combined with advanced search engines and big-data platforms, this pipeline supports early biomarker identification and targeted therapies, and remained helpful for longitudinal disease monitoring and personalized treatment adjustments.

A deeper understanding of proteomic changes and PTMs in ocular fluids could facilitate the identification of novel molecular biomarkers, enable more precise risk stratification, and support the development of tailored therapies using novel therapeutic targets. However, while ocular fluids such as aqueous and vitreous humor are promising sources for biomarker discovery, comparative proteomic studies have shown that they only partially reflect the retinal proteome, likely due to physiological barriers and tissue compartmentalization (Grus et al., 2019).

## Conclusions

Advanced mass spectrometry-based proteomic workflows are expanding our understanding of the retinal proteome in both health and disease. The identification of new differentially expressed proteins in various ocular and neurodegenerative diseases with retinal manifestations holds promise as potential therapeutic targets and diagnostic biomarkers that rely on the retina. While transitioning from retinal biomarkers to blood-based detection systems remains a challenging task due to the complex correlation between these two proteome sets, the retina is still the main source of sampling for routine analytic procedures. Retinal proteome studies continue to offer significant potential for elucidating disease mechanisms, identifying drug targets, and developing novel treatments or neuroprotective strategies. As high-dimensional proteomic datasets expand, advanced artificial intelligence architectures ranging from deep neural networks to transfer learning offer unprecedented opportunities to decode the intricate molecular interactions underlying retinal neurodegeneration and gain a better understanding of the pathophysiological mechanisms underlying these diseases. Novel affinity proteomic technologies, while underutilized, hold great promise for advancing retinal-based proteomics, as these technologies can deliver large proteome coverage at a wide dynamic range using minimal sample loading.

## Data Availability

*The mass spectrometry proteomics data on human AD brains and retinas have been deposited to the Proteome Xchange Consortium via the PRIDE partner repository with the dataset identifier PXD040225. Additional data are available from the corresponding author upon reasonable request.*
